# Dietary inulin supplementation modulates the composition and activities of carbohydrate-metabolizing organisms in the cecal microbiota of broiler chickens

**DOI:** 10.1371/journal.pone.0258663

**Published:** 2021-10-21

**Authors:** Yun Xia, Jiarong Miao, Yu Zhang, Hongbo Zhang, Lingdong Kong, Robert Seviour, Yunhong Kong

**Affiliations:** 1 School of Agriculture and Life Science, Kunming University, Kunming, China; 2 First Affiliated Hospital of Kunming Medical University, Kunming, China; 3 Microbiology Department, La Trobe University, Bundoora, Victoria, Australia; 4 Dianchi Lake Environmental Protection Collaborative Research Center, Kunming University, Kunming, China; Wageningen Universiteit, NETHERLANDS

## Abstract

Inulin is a highly effective prebiotic and an attractive alternative to antibiotic growth promoters for increasing production and maintaining health in chickens. However, how inulin elicits its effects on members of the intestinal microbiota is unknown, even though their importance for energy metabolism and the health of chickens is well documented. A combination of 16S rRNA Illumina sequencing and transcriptomic analysis was used to investigate the effects of supplementing a corn-based basal diet with 1, 2, or 4% inulin or 400 ppm bacitracin on the composition, diversity and activities of carbohydrate-metabolizing organisms (CMOs) in the cecal microbiota of broiler chickens. We found that members of *Bacteroides* were the most abundant non-starch degrading CMOs, contributing 43.6–52.1% of total glycoside hydrolase genes and 34.6–47.1% activity to the meta-transcriptomes of chickens in the different dietary groups, although members of *Parabacteroides*, *Prevotella*, *Alistipes*, *Clostridium*, *Barnesiella*, *Blastocystis*, *Faecalibacterium* and others were also actively involved. Inulin and bacitracin inclusion in the basal diet did not change significantly the composition or diversity of these CMOs. Inulin supplementation at three levels promoted the activities of *Bacteroides*, *Prevotella* and *Bifidobacterium*, and 2% level appears to be the most optimal dosage for bifidobacterial activity.

## Introduction

Inulin is a highly effective prebiotic with potential as an alternative to the antibiotic growth promoters currently used in the livestock industry to increase animal productivity and maintain their health [1]. Inulin consists of two fructose subunits linked by β-1,2 bonds, with a single molecule of glucose at the reducing end linked by an α-1,2 linkage to a fructose residue. The β-1,2 linkages protect inulin from enzymatic digestion in the upper gastrointestinal tract [2], allowing it to reach the cecum unmodified, where it is then degraded and further fermented to short-chain fatty acids (SCFAs) by the cecal microbiota [3]. These SCFAs can enter the bloodstream and contribute partially (5–10%) to the energy requirements of chickens. The presence of SCFAs may also decrease the cecal pH, resulting in pathogen inhibition, decreased bile acid solubility, an indirect increase in mineral absorption, and reduced ammonia absorption by protonic dissociation of ammonia and other amines [4].

Dietary inulin inclusion has been reported to improve growth performance of chickens [5,6] and pigs [7–9], to enhance their immune system function [10,11], and to increase their lipid and cholesterol metabolism [12,13]. In ovo inulin delivery is thought to extend the lifespan of chickens by stimulating the colonization of their embryonic gastrointestinal tract by native microbiota, thereby facilitating the establishment of an optimized microbiome [14]. However, results from studies on how dietary inulin supplementation affects growth performance have been inconsistent, with positive impacts appearing to depend on the source and inclusion level of inulin in the basal diet (BD), individual animal attributes, and levels of animal hygiene [15]. In some studies, inulin supplementation had no effect on chicken growth performance [11,16], and even where improvement in growth performance was recorded, the mechanisms involved have remained largely unknown [1].

Prebiotic effects of inulin on chickens are thought to result from alterations to gut microbiota composition and their metabolic activity [1]. The cecal microbiota play important roles in minimizing pathogen colonization, detoxification of potentially harmful substances, recycling of nitrogen compounds, degradation and absorption of nutrients, and stimulation of host metabolism and immunological activity [17,18]. However, most previous such studies have used culture-dependent techniques [19–22], with their widely known limitations in revealing the level of microbial diversity in complex ecosystems such as the cecal microbiota by comparison with high throughput sequencing. Although inulin supplementation has been reported to stimulate the growth of the bacteria *Bifidobacterium* [5,19–23] and *Lactobacillus* [5,19,23], not all published data are in agreement [2,24]. Furthermore, little is known about the fate of inulin and how inulin might affect carbohydrate metabolism there.

We have used 16S rRNA Illumina sequencing combined with transcriptomic analysis to investigate the effects of dietary inulin supplementation on the phylogenetic composition and diversity and activities of carbohydrate-metabolizing organisms (CMOs) in chicken cecal microbiota. The information generated will improve our understanding of the mode of action of inulin, and lead to improved inulin feeding regimes optimizing its prebiotic impact.

## Materials and methods

### Ethics approval

Animal care and experimental protocols (YAG2017015) were approved by the Animal Care and Use Committee of Yunnan Agriculture University, China. The animals were cared for according to the Animal Care Guidelines of China. All efforts were made to minimize animal suffering.

### Animal experimental design

Thirty-five one-day-old male Tegel broiler chicks from the same parent flock (age 140 days) were obtained from a local commercial hatchery and randomly divided into 5 dietary groups of 7 chickens each based on similar body weight (46 ± 5 g). The five dietary treatments consisted of the basal diet (BD) control, the BD plus 400 ppm bacitracin, and the BD plus 1% inulin (10 g inulin/kg), 2% inulin (20 g inulin/kg), or 4% inulin (40 g inulin/kg). Each group of 7 chickens was housed in a cage with 1.5 m^2^ floor area. The chickens were fed with a corn-based “starter” diet from 1 to 21 days and a “grower” diet from 22 to 42 days, their normal commercial lifespan. Starter and grower diets (Table 1) were formulated to meet the nutritional requirements of chickens as recommended by the NRC [25]. Bacitracin (10% active ingredient content) was purchased from a local commercial poultry antibiotic supplier (Lukang Biological Manufacture Co., Shandong, China). Inulin derived from chicory roots and with a polymerization degree between 10 and 60 was purchased from Orafti GR (BENEO-Orafti B 3300, Tienen, Belgium). Inulin and bacitracin were supplied in powder form and mixed with the BD to the designated concentrations through replacement of the same quantity of corn powder. Chickens were housed in environmentally controlled conditions. Diets were offered twice daily (8:00 and 18:00) and water was provided *ad libitum*. The housing temperature was maintained at 34°C for the first 5 days and gradually decreased to 24°C, which was maintained until the end of the experiment. Continuous lighting was provided throughout the experiment.

**Table 1:** Composition of the experimental diet (%).

Ingredient	Starter (0-21d)	Grower (22-42d)
Corn (%)	58	61.8
Soybean meal (%)	27	23.7
Corn gluten meal (%)	5	6.9
Fish meal (%)	2.8	0
Soybean oil (%)	3	3.2
Calcium hydrogen phosphate (%)	1.45	1.68
Fine stone powder (%)	1.15	0.76
Coarse stone powder (%)	0	0.4
Salt (%)	0.23	0.33
Methionine (%)	0.17	0.06
Lysine (%)	0.16	0.17
Premix* (%)	1	1
Total	100	100
**Calculated nutrition composition**		
ME (KC/kg)	3050	3100
CP (%)	22	20
Ca (%)	1	1.06
P (%)	0.7	0.74
Zn (mg/kg)	185	210
Fe (mg/kg)	337	404
Mn (mg/kg)	226	278
Mg (%)	0.17	0.19
Cu (mg/kg)	59	70.5
Na (%)	0.24	0.34
K (%)	0.81	0.72
AP (%)	0.45	0.4
NaCl (%)	0.35	0.35
Crude protein (%)	23.6	22.6
Crude fiber (%)	2.7	2.33
Crude fat (%)	4.82	4.49
Dry matter (%)	89.2	89.9
Tryptophan (%)	0.26	0.23
Aspartic acid (%)	1.61	1.79
Threonine (%)	0.71	0.79
Serine (%)	0.95	1.04
Glutamic acid (%)	3.65	4.26
Glycine (%)	0.76	0.79
Alanine (%)	0.82	0.97
Cysteine	0.36	0.39
Valine (%)	0.53	0.57
Methionine (%)	0.33	0.33
Isoleucine (%)	0.79	0.88
Leucine (%)	2.15	2.52
Tyrosine (%)	0.61	0.76
Phenylalanine (%)	0.98	1.08
Histidine (%)	0.45	0.44
Lysine (%)	1.14	1.17
Arginine (%)	1.02	1.1
Proline (%)	1.13	1.1

*2.5 kg of vitamin premix contains: 10.8 g retinal, 1.6 g calcidiol, 72 g tocopheryl acetate, 8 g menadione, 7.2 g thiamine, 26.4 g riboflavin, 40 g niacin, 120 g calcium pantothenate, 12 g pyridoxine, 4 g folic acid, 0.06 g cyanocobalamin, 1000 g choline chloride, 0.4 g biotin.

### Sample collection

Chickens were chosen randomly from each treatment group at the end of the experiment (day 42), injected intravenously with sodium pentobarbital (50 mg/kg), and immediately necropsied to harvest cecal samples. For RNAseq analysis, cecal samples (*n* = 3) from each treatment group were removed aseptically and immediately immersed in liquid nitrogen for subsequent determination of mRNA expression. For analyses of microbial composition and biodiversity, aseptically removed cecal samples from each treatment group (*n* = 4) were frozen at −80°C for 16S rRNA gene sequencing. The sample size was determined with reference to the ARRIVE guideline (https://arriveguidelines.org/), with an experimental unit of a single animal.

### 16S rRNA amplicon library preparation and sequence analyses

16S rRNA amplicon library preparation and sequence analyses were carried out according the methods described in Xia et al. [26]. Briefly, total genomic DNA was extracted using the Qiagen QIAamp Fast Stool Mini Kit (Qiagen, Shanghai, China) according to the manufacturer’s protocol. DNA concentration was determined using a Nano-Drop 2000 spectrophotometer. The V3-V4 hypervariable regions of the bacterial 16S rRNA gene were amplified in triplicate using the primer pair 338F (5ʹ-ACTCCTACGGGAGGCAGCAG-3ʹ) and barcoded 806R (5ʹ-GGACTACHVGGGTWTCTAAT-3ʹ) [27] under the following PCR cycling conditions: initial denaturation at 94°C for 3 min followed by 5 cycles of denaturing at 94°C for 10 s, annealing at 55°C for 15 s, and extension at 72°C for 30 s before a final extension at 72°C for 5 min. PCR products were visualized on 2% agarose gel (in TAE buffer) containing ethidium bromide and purified with a DNA gel extraction kit (Axygen, Shanghai, China). Sequencing was performed on a HiSeq 2000 equipment at the Majorbio Bio-Pharm Technology Co., Ltd., Shanghai, China.

The V3-V4 amplicons of 16S rRNA genes were pair-end assembled and checked using the software Flash [28] to ensure that their sequences matched perfectly with those of the index sequences, had no more than one 1 mismatch error present in the forward primer sequences, and trimmed sequences were longer than 200 bp. Trimmed high-quality sequences of each bacterial community were uploaded into QIIME [29] to perform operational taxonomic unit (OTU) clustering, alpha and beta diversity analyses after standardization of the numbers of 16S rRNA reads to their minimum of 18,517. The V3-V4 amplicon sequences were grouped into OTUs at the 97% identity threshold (3% dissimilarity levels) using RDP classifier (Release 11.1 hppt://rdp.cme.msu.edu/). Any OTU represented by ≤1 sequences was removed. The 16S rRNA sequences have been deposited in the NCBI Sequence Read Archive under Submission ID: PRJNA523884 (https://www.ncbi.nlm.nih.gov/bioproject/PRJNA523884/).

### RNA extraction, library preparation, and sequencing

RNA extraction, library preparation, and sequencing were carried out according to the methods described by Xia et al. [26]. Briefly, total RNA was isolated using TRIzol reagent (Thermo Fisher Scientific, Shanghai, China) after grinding the frozen cecal sample to a fine powder in liquid nitrogen. To identify the CMOs in the cecal microbiota following dietary inulin supplementation, equimolar amounts of RNA extracted from 3 chickens of each group were pooled for transcriptomic analysis. The rationale for pooling RNA samples from individual samples was that it is cost effective and provides genome-wide information about potentially functionally relevant variations [30]. The purpose of the RNA-seq analyses performed here was to elucidate changes in the expression of genes involved in metabolic pathways as a result of dietary supplementation with inulin. The quality and quantity of extracted RNAs were monitored on 1% agarose gels before rRNAs were removed using Ribo-Zero rRNA Removal Kits (Qiagen, Shanghai, China) following the manufacturer’s instructions. For each sample, a library with about 200 bp insert sizes was prepared with a TruSeq RNA Sample Prep Kit (Qiagen, Shanghai, China), and mRNAs were amplified with a “bridge PCR” using a HiSeq 3000/4000 Cluster Kit (Illumina, Shanghai, China) according to the manufacturer’s instructions. The cDNAs obtained were subjected to 2 × 100 bp paired-end (PE100) sequencing on a HiSeq 2000 instrument using HiSeq 3000/4000 SBS Kits (Illumina, Shanghai, China) at the HiSeq platform of Majorbio Bio-Pharm Technology Co., Ltd., Shanghai, China. The raw RNA sequence dataset supporting the conclusions of this article is available in the NCBI Short Reads Archive (SRA) under the submission ID PRJNA523864 (https://www.ncbi.nlm.nih.gov/bioproject/?term=PRJNA523864).

### Sequence quality control and genome assembly

The cDNA sequences at 3`and 5`ends were truncated using SeqPrep (https://github.com/jstjohn/SeqPrep) and cleaned of adapter contamination, or at least 10 Ns from the raw data (FASTQ format) using Sickle (https://github.com/najoshi/sickle) and assembled using SOAPdenovo (http://soap.genomics.org.cn, Version 1.06). Scaffolds with a length over 500 bp were extracted and broken into contigs without gaps. Contigs were used for further gene prediction and annotation.

### Gene prediction, taxonomy and functional annotation

Gene prediction, taxonomy and functional annotations were carried out according the method protocols described by Xia et al. [26]. Briefly, open reading frames (ORFs) from each cecal sample were predicted using MetaGene (http://metagene.nig.ac.jp/metagene/download_mga.html). The predicted ORFs with lengths of more than 100 bp were retrieved and translated to amino acid sequences using the NCBI translation table (http://www.ncbi.nlm.nih.gov/Taxonomy/taxonomyhome.html/index.cgi?chapter=tgencodes#SG1). All sequences from gene sets with a 95% sequence identity (90% coverage) were clustered as a non-redundant gene catalog by the CD-HIT (http://www.bioinformatics.org/cd-hit/). After quality control, reads were mapped to the representative genes with 95% identity using SOAPaligner (http://soap.genomics.org.cn/), and gene abundances in each sample were evaluated. BLASTP (Version 2.2.28+, http://blast.ncbi.nlm.nih.gov/Blast.cgi) was employed for taxonomic annotations by aligning non-redundant gene catalogs against NCBI NR database with e-value cutoff of 1e^-5^. Cluster of orthologous groups of proteins (COG) for the ORFs annotation was performed using BLASTP against eggNOG database (v4.5) with an e-value cutoff of 1e^-5^.

### Carbohydrate-active enzymes annotations

Carbohydrate-active enzyme annotations were conducted using hmmscan (http://hmmer.janelia.org/search/hmmscan) against the CAZy database V5.0 (http://www.cazy.org/) with an e-value cutoff of 1e^-5^.

### Statistical analyses

Rarefaction analyses were performed and biodiversity indices including the Chao1 index, Shannon index, coverage ratios were calculated with Mothur [31] following the procedures provided, and applying a 97% identity threshold. Significant differences in relative percentage abundance at phylum and genus levels between the cecal microbiota of chickens fed the different diets were determined using Kruskal Wallis test. Significant differences were established at *P* < 0.05. Principal coordinate analyses (PCoA) and Venn diagrams of the OTU distribution in each cecal microbiota were drawn using R software (https://www.r-project.org). PERMANOVA analyses were performed (999 permutations) on the Bray-Curtis matrix. Significant differences were established at *P* < 0.05. All these statistical analyses were performed with the platform of Cloud Majorbio (https://cloud.majorbio.com).

## Results

### Effects of dietary inulin and bacitracin supplementation on the diversity and composition of the cecal microbiota

**Fig 1:**
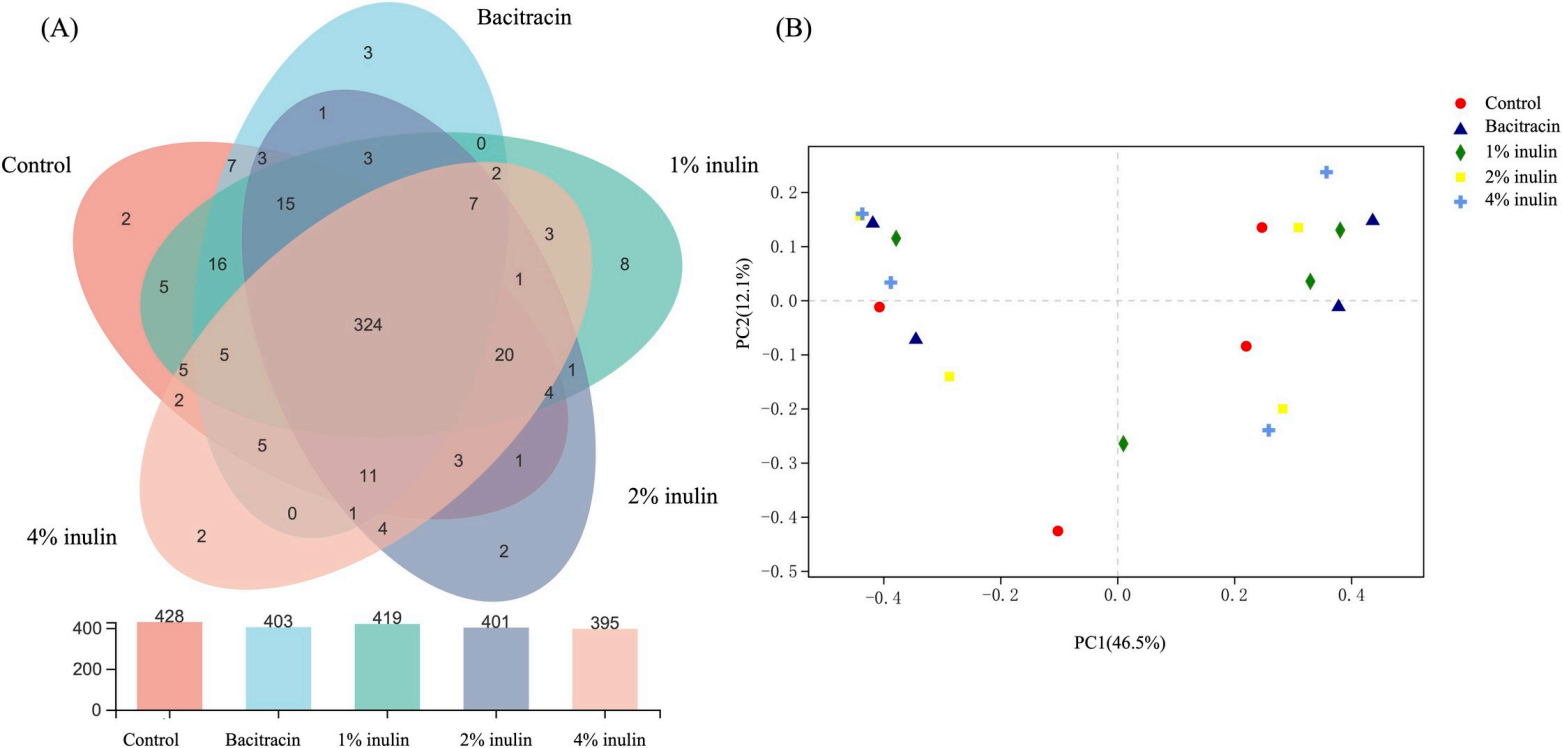
Effects of dietary supplementation with bacitracin and inulin on the distribution of bacterial OTUs and community composition in the cecal microbiota of broiler chickens. **(A)** Venn diagram showing the occurrence of bacterial OTUs identified in 16S rRNA sequencing of cecal microbiota of chickens. **(B)** Grouping of cecal bacterial communities based on principle component analyses of Illumina sequencing of 16S rRNA amplicons (V3-V4 region).

The 16S rRNA gene amplicon sequences from the ceca of chickens fed the diets described above were compared to determine their effects on the corresponding community diversity and compositions. In total, 510,382 bacterial 16S rRNA high-quality reads were obtained from the 20 (four replicates for each treatment) cecal DNA samples obtained from chickens fed with a BD, BD plus 400 ppm bacitracin, and the BD plus 1% inulin, 2% inulin, or 4% inulin. A total of 466 bacterial operational taxonomic units (OTUs) (Fig 1A) belonging to at least 11 phyla and 122 genera (S1 Table) were identified. Of these, 324 OTUs were shared by all cecal microbiota with different dietary treatments (Fig 1A). The rarefaction curves based on Chao1 and Shannon indices and Coverage values (S1 Fig) showed that the selected sequencing depths covered adequately the microbial diversity in the microbiota samples.

**Fig 2:**
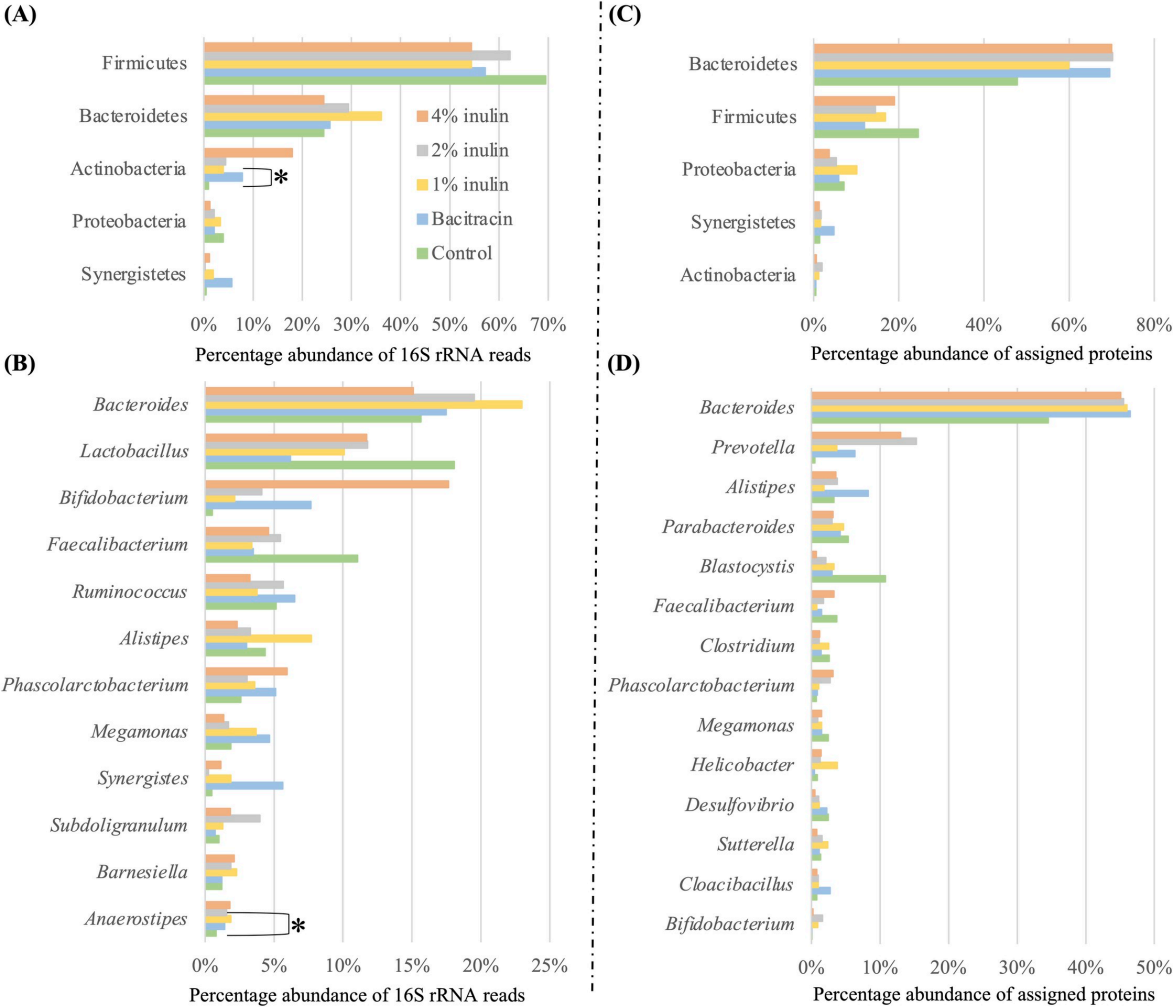
Phylum (A) and genus (B) compositions of the bacterial communities in the cecal microbiota characterized based on 16S rRNA amplicons (V3-V4 region) sequencing. Relative expression abundance of assigned proteins at phylum (C) and genus (D) level based on transcriptome analysis of the cecal microbiota. Symbol labels in (A) apply to (B), (C) and (D). * significant (*P* < 0.05) difference between two dietary groups.

Members of phyla Firmicutes and Bacteroidetes (Fig 2A), and genera *Bacteroides*, *Lactobacillus*, *Bifidobacterium*, *Faecalibacterium*, *Ruminococcus*, *Alistipes*, *Phascolarctobacterium*, *Meganomas*, *Synergistes*, *Subdoligranulum*, *Barnesiella* and *Anaerostipes* (Fig 2B) constituted a major fraction of the cecal microbiota developed with the different dietary treatments. Dietary supplementation with bacitracin and different concentrations of inulin did not significantly (*P* > 0.05) affect the percentage abundances of total 16S rRNA reads (abbreviated as 16S rRNA abundances) of most of these genera (S2 Fig). Dietary supplementation with 1% inulin significantly (*P* = 0.02) increased 16S rRNA abundances of members of the Actinobacteria (Fig 2A). At genus level, supplementation with inulin increased significantly (*P* < 0.05) 16S rRNA abundances of some less dominant genera, including members of *Flavonifractor* with 1% inulin (S2 Fig), those of *Anaerostipes* with 2% inulin (S2 Fig), and members of *Anearofilum* with 4% inulin (S2 Fig). Bacitracin supplementation did not significantly affect (*P* > 0.05) 16S rRNA abundances of any of these genera (S2 Fig). PCoA analyses revealed no clear clustering patterns among the four-replicate microbiota in the same dietary treatment group (Fig 1B) and nor did PERMANOVA analyses reveal any significant differences (*P* = 0.946) among the cecal microbiota of chicken fed different dietary treatments.

### Effects of inulin and bacitracin dietary inclusion on the gene function and gene expression abundance of the cecal microbiota

**Fig 3:**
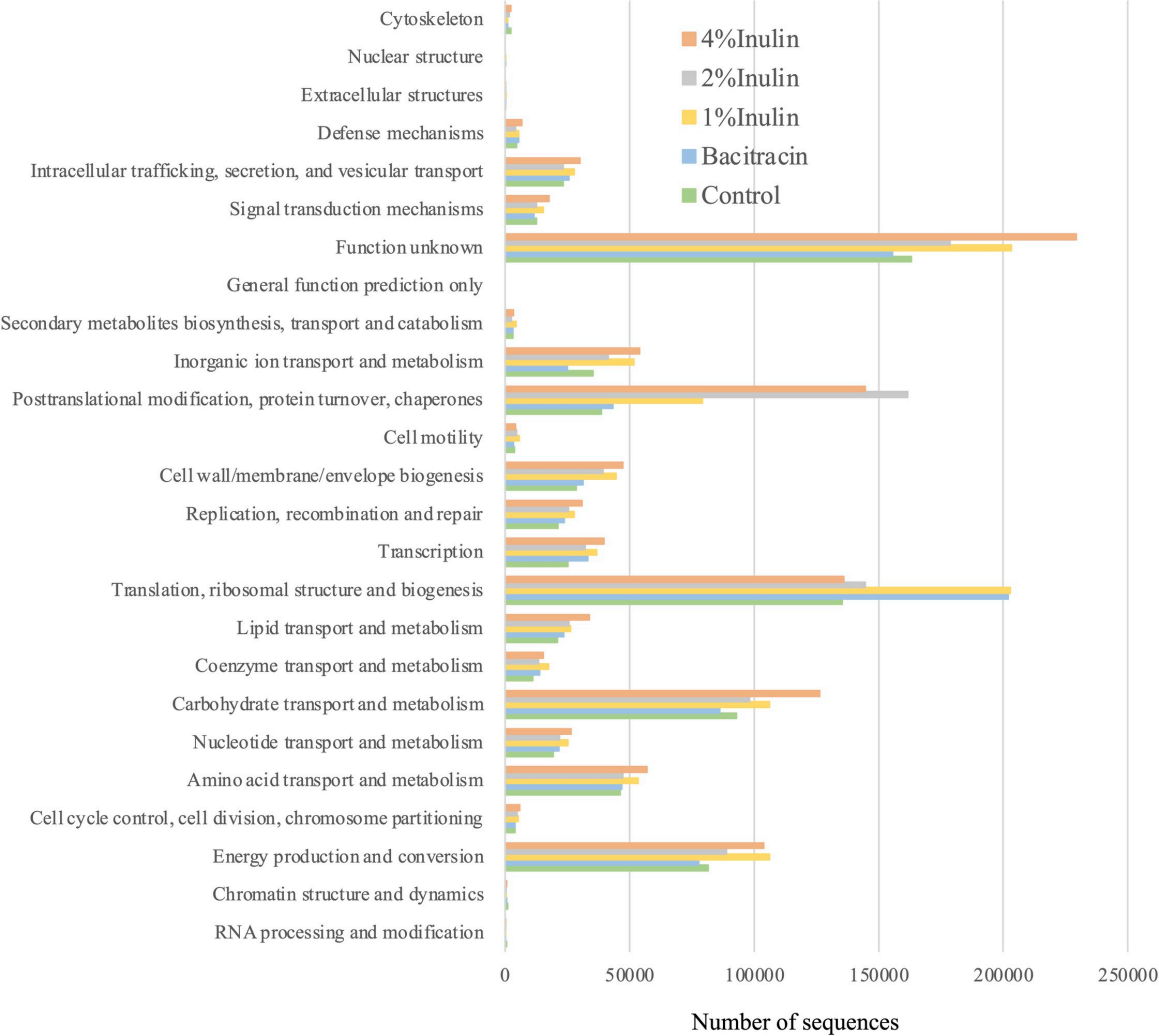
Expression numbers of functional genes based on cluster of orthologous groups of proteins identified in the cecal microbiota of broilers fed a basal diet supplemented with 0 (control), 1%, 2% or 4% inulin or 400 ppm bacitracin.

Each cecal RNA sample yielded between 55,069 to 80,389 ORFs useable for gene expression analysis. Expression of the unigenes in each cecal sample classified according to their COG function are shown in Fig 3. The inulin diet supplement at the three levels increased to differing degrees the expression numbers of functional genes (Fig 3). Thus, by comparison with those expressed in the cecal microbiota of the control group, cecal microbiota of chickens supplemented with inulin feed expressed higher levels of the genes encoding polypeptides associated with translation, ribosomal structure and biogenesis, carbohydrate transport and metabolism, energy production and conversion, posttranslational modification, protein turnover, and chaperones, and others. In contrast, bacitracin supplementation increased the expression of genes encoding the polypeptides associated with translation and transcription, ribosomal structure and biogenesis, and coenzyme transport and metabolism.

Bacteroidetes members contributed an average of 63.5% metabolic activity in all cecal meta-transcriptomes of chicken in different dietary treatment groups (Fig 2C) followed by members of the Firmicutes, Proteobacteria, Synergistetes, and Actinobacteria. Compared to the control diet fed chickens, dietary supplementation with 1%, 2%, and 4% inulin increased the metabolic activity from members of the Bacteroidetes, Actinobacteria, and decreased that of Firmicutes. Inclusion of bacitracin in the BD increased the metabolic activity of members of the Bacteroidetes, Synergisteres, and Proteobacteria, but decreased that from members of the Firmicutes (Fig 2C).

At the genus level (Fig 2D), members of the *Bacteroides* contributed an average of 43.6% metabolic activity in all the cecal meta-transcriptomes of chicken fed with different dietary treatments, followed by *Prevotella*, *Alistipes*, *Parabacteroides*, *Blastocystis*, *Faecalibacterium*, and others (Fig 2D). Dietary supplementation with 1, 2, and 4% inulin increased the metabolic activity from *Bacteroides*, *Prevotella*, *Helicobacter*, and *Phascolarctobacterium*, but decreased that from *Blastocystis*, *Parabacteroides*, *Clostridium*, *Desulfovibrio* and *Megamonas* (Fig 2D). Supplementation with bacitracin increased the metabolic activity from the genera *Bacteroides*, *Prevotella*, and *Alistipes*, but decreased that of *Blastocystis*, *Parabacteroides*, *Faecalibacterium*, *Clostridium* spp. and *Megamonas* (Fig 2D).

### Expression of genes encoding carbohydrate metabolism-active enzymes in the cecal microbiota of chickens treated with inulin and bacitracin

**Fig 4:**
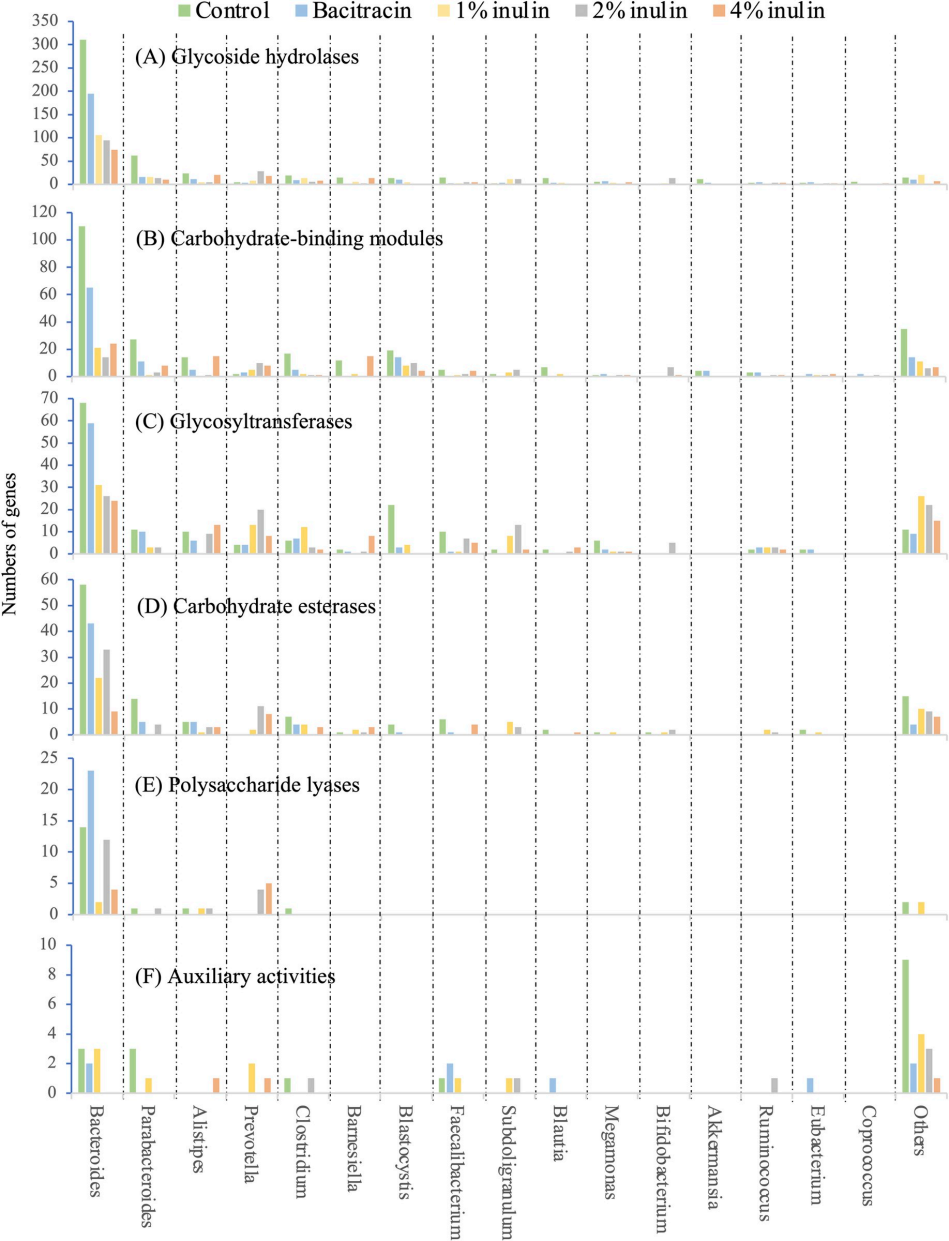
Phylogenetic composition of carbohydrate metabolizing genes and their expression numbers in the cecal microbiota of broilers fed a basal diet supplemented with 0 (control), 1%, 2% or 4% inulin or 400 ppm bacitracin.

To identify the CMOs responsible for the degradation of non-starch polysaccharides, especially inulin, we analyzed the taxonomic distribution of genes coding for glycoside hydrolases (Fig 4A), carbohydrate-binding modules (Fig 4B), glycosyltransferases (Fig 4C), carbohydrate esterases (Fig 4D), polysaccharide lyases (Fig 4E), and auxiliary activities (Fig 4F) using the CAZy database. We found that members of the genera *Bacteroides*, *Parabacteroides*, *Alistipes*, *Clostridium*, *Barnesiella*, *Faecalibacterium*, *Blautia*, *Blastocystis*, *Akkermansia*, *Megamonas*, and *Coprococcus* together expressed 94.8% of the classifiable glycoside hydrolases-encoding genes (Fig 4A), 77.1% of the carbohydrate-binding modules-encoding genes (Fig 4B), 82.9% of the glycosyltransferases-encoding genes (Fig 4C), 78.5% of the carbohydrate esterases-encoding genes (Fig 4D), 84.2% of the polysaccharide lyases-encoding genes (Fig 4E), and 41.2% of the auxiliary activities-encoding genes (Fig 4F) in the cecal microbiota of the control group, indicating that these populations were the main CMOs in the chicken ceca.

The distribution of the glycoside hydrolases family 32 (abbreviated as GH32 hereafter)-encoding genes (http://www.cazy.org/GH32.html) and their expression number in the cecal microbiota of chickens fed diets supplemented with or without inulin was also investigated to identify the potential inulin-hydrolyzing organisms. The GH32 enzymes include endo- and exo-inulinase (http://www.cazy.org/GH32.html) able to hydrolyze β-1,2 glycosidic linkages to produce fructose, inulo-oligosaccharides, and glucose [32]. In total, 68 such genes were traced here to members of the *Bacteroides*, *Prevotella*, *Eubacterium*, *Barnesiella*, *Bifidobacterium*, *Clostridium*, *Acholeplasma*, *Faecalibacterium*, *Blautia*, *Lactobacillus*, and unclassified bacteria (S3 Table). Compared with the control diet group, the numbers of genes encoding the GH32 enzymes from *Bacteroides* (after exposure to 1 and 2% inulin), *Prevotella* (after exposure to 1, 2 and 4% inulin), unclassified Firmicutes (after exposure to 1, 2 and 4% inulin), unclassified *Lachnospiraceae* (after exposure to 1% inulin), unclassified *Ruminococcaceae* (after exposure to 1 and 2% inulin), *Barnesiella* (after exposure to 1 and 2% inulin), *Bifidobacterium* (after exposure to 1 and 2% inulin), *Clostridium* (after exposure to 4% inulin) all increased (Fig 4). Thus, these bacteria were most probably those involved in inulin hydrolysis. In contrast, dietary supplementation with 400 ppm bacitracin did not markedly affect the expression levels of these GH32-encoding genes in any population, as expected.

## Discussion

Dietary supplementation with inulin has been reported to enhance immune system efficiency [10,11] as well as promoting lipid and cholesterol metabolism [12,13] in livestock animals, and consequently to improve their growth performance [5–9]. These beneficial effects are thought to be exerted through modulation of the gut microbiota and their relative expressions of key catabolic enzymes [1]. In this study, we have used 16S rRNA gene sequencing in combination with transcriptomic analysis to investigate the effects of the dietary treatments with 1%, 2% and 4% inulin and 400 ppm bacitracin on both the composition, diversity and key metabolic features of their cecal microbiota. Bacitracin, used extensively as an antibiotic growth promoter to improve poultry productivity [33] was used as the antibiotic control. Bacitracin is a mixture of high molecular weight polypeptides that possess antimicrobial activity predominantly against Gram-positive bacteria, by interfering with bacterial cell wall peptidoglycan biosynthesis [34].

The gut microbiota able to degrade metabolically recalcitrant carbohydrates are more likely to mediate beneficial effects of dietary supplementation with inulin, because most of the more readily metabolizable substrates in chicken diets are metabolized earlier in their passage through the gastrointestinal system [2]. We have shown here with CAZy analysis that *Bacteroides* spp. were the most abundant non-starch degrading CMOs in the ceca of chickens fed a corn-based basal diet by contributing 43.6–52.1% of total glycoside hydrolase encoding genes (S1 Fig) and 34.6–47.1% metabolic activity to the meta-transcriptomes of chickens in different dietary groups, although members of *Parabacteroides*, *Alistipes*, *Clostridium*, *Barnesiella*, *Blastocystis*, *Faecalibacterium*, *Subdoligranulum*, *Blautia*, *Akkermansia*, and *Megamonas* appeared to be involved.

Similar studies have shown that dietary inulin supplementation leads to an enrichment of *Bifidobacterium*, and sometimes *Lactobacillus* in both the poultry [5,19–21,23] and human ceca [35]. Thus, these bacteria are probably universally those most responsible for any probiotic effect inulin might have [36]. Members of both genera appear to satisfy the criteria for probiotic agents, based on their immuno-stimulatory properties, together with their ability to outcompete pathogenic bacteria for cell adhesion sites, and their abilities to generate volatile fatty acids for host energy production (reviewed by Adhikari and Kim, [37]; Binda et al. [38]). However, to the best of our knowledge, the mechanism(s) responsible for this bifidogenic effect of inulin in chickens has/(ve) never been clarified previously. We show here that supplementation with inulin at levels of 1%, 2% and 4% had no significant effect on the 16S rRNA abundances of these two populations. However, *Bifidobacterium* populations responded positively to the presence of inulin by an increase in their GH32 enzyme activity compared to that in the diet group (no activity detected) to 1.8%, 7.7% and 0.25% with 1%, 2% and 4% inulin dietary inclusion (S3 Table) confirming its involvement in inulin metabolism. Furthermore, inulinase activities has been reported in cultured strains of *Bifidobacterium* [3]. In contrast, *Lactobacillus* appear to play only a minor role in inulin hydrolysis (Fig 4), even though some strains (e.g. *L*. *paracasei*) are known to exhibit exo-inulinase activity, especially against short-chain inulin substrates with low degrees of polymerization of 3 and 5 [39]. Long-chain inulin (degrees of polymerization 10–60) was used in our feed experiments.

Our transcriptomic data also show that, in addition to *Bifidobacterium*, members of *Bacteroides*, *Prevotella*, *Barnesiella*, *Clostridium* and other as yet unclassified bacteria in chicken cecal microbiota responded positively to inulin, since as the expression levels of their GH32-encoding genes also increased (S3 Table). Therefore, these too may play a role in inulin hydrolysis. Moreover, with the exception of *Prevotella*, which showed an increase in expression levels of their GH32-encoding genes in response to all three inulin levels (Fig 4), these other putative inulin-hydrolyzing organisms showed a dose-dependent response. For example, *Bacteroides* and *Barnesiella* in response to 1% and 2% inulin and *Clostridium* in response to 4% inulin only. *Bifidobacterium* spp. had the highest GH32 enzyme (S3 Table) and total enzyme expression levels (S2 Table) in response to 2%. Such data provide for the first-time metabolic evidence demonstrating that inulinase is almost certainly an inducible enzyme where inulin levels affect the corresponding expression levels of inulinase encoding gene/s. This dose dependent effect on inulinase expression may help to explain the conflicting published results concerning its impact on growth performance parameters in livestock animals.

However, based on our 16S rRNA gene amplicon sequencing data, no *Prevotella* members were detected in any of these cecal communities. This trend could reflect a bias associated with PCR amplification (i.e., suboptimal PCR primers or targeted rRNA variable region) and/or the small number (*n* = 4) of cecal samples sequenced for each dietary treatment group. Furthermore, inulin exposure also led to a concentration-dependent increase in the 16S rRNA abundance of *Prevotellaceae* UCG-001 family members, from non-detectable to between 0.8% and 1.6% abundance (S2 Table). These data suggest that the role in inulin and polysaccharide utilization attributed here to *Prevotella* could have instead involved other *Prevotellaceae* UCG-001 family members. Importantly, transcriptomic analysis by Song et al. [40] indicated that supplementation with 3% inulin substantially enriched the *Prevotellaceae* UCG-001 members in the cecal microbiota of *ob*/*ob* mice. Moreover, the 16S rRNA abundance of these *Prevotellaceae* correlated positively with an up-regulation in the mouse liver of the AMP-activated protein kinase signaling pathway, an enzyme with an essential role in controlling energy balance in its animal host [41].

Taken together, our transcriptomic and 16S rRNA data have revealed that potentially pathogenic populations, including those of pathogenic *Escherichia* spp., contributed only a minor fraction of the cecal microbiota of chicken in all dietary groups, and their 16S rRNA abundances (S1 Table) and metabolic activity (S2 Table) did not change substantially with dietary inulin or bacitracin supplementation. However, transcriptomic data showed that the pathogenic unicellular protozoan *Blastocystis hominis* was present and actively involved in cecal metabolism. This species comprises at least 17 subtypes and is present in the intestines of chickens, human and other animals [42]. Very little is known of its basic biology, and controversy surrounds its taxonomy and pathogenicity [43]. In our study, *B*. *hominis* contributed 10.8% metabolic activity of the cecal meta-transcriptome of chickens in the control group (S2 Table), including those of glycoside hydrolases- (Fig 4A), carbohydrate-binding modules- (Fig 4B), glycosyltransferases- (Fig 4C), and carbohydrate esterases-activity (Fig 4D). These data suggest that *B*. *hominis* is involved in active cecal degradation of non-starch polysaccharides, and/or in attacking chicken intestinal epithelial cells, as has been suggested by Denoeud et al. [44]. Although glycoside hydrolases-encoding genes have been identified in the genome of *Blastocystis* strains (ST3) [45], to the best of our knowledge, this is the first report of *Blastocystis* exhibiting glycoside hydrolases-, carbohydrate-binding modules-, glycosyltransferases-, and carbohydrate esterases-activity in chicken ceca. Here, we show for the first time that dietary supplementation with 4% inulin markedly inhibited the gene expression of these enzymes (Fig 4) and the metabolic activity (S2 Table) in *Blastocystis*. This suggests that inulin has potential as an alternative to currently used anti-*Blastocystis* drugs, and is safer, healthier, and cheaper.

Our meta-transcriptomic and 16S rRNA data are in agreement, with both showing that members of genera *Bacteroides*, *Prevotella*, *Alistipes*, *Parabacteroides*, *Faecalibacterium*, *Bifidobacterium*, *Clostridium*, *Phascolarctobacterium*, *Megamonas*, *Helicobacter*, *Desulfovibrio*, *Sutterella*, *Cloacibacillus*, *Methanocorpusculum*, and *Barnesiella* are the main microorganisms present in broiler cecal microbiota. Those of *Bacteroides*, *Parabacteroides*, *Prevotella*, *Alistipes*, *Clostridium*, *Barnesiella*, *Blastocystis*, *Faecalibacterium* are the main non-starch degrading CMOs, while members of *Bacteroides*, *Prevotella*, *Bifidobacterium*, *Barnesiella*, *Clostridium* are the main inulin-hydrolyzing organisms. Of them, members of the genus *Bacteroides* should be viewed as the key populations responsible both quantitatively and qualitatively for many of the crucial metabolic transformations. Inulin supplementation at 2% level appears to be the most optimal dosage for bifidobacterial activity. However, we realized that this experiment was performed with a single animal and with limited numbers of birds used in individual treatment groups. In addition, no negative or positive controls were included in the 16S rRNA library constructions, which could affect accurate determinations of the abundances of the less abundant bacterial populations.

## Supporting information

S1 Fig Rarefaction curves for OTU (Chao1), Shannon index (B) and Coverage ratios (C) calculated using Mothur (v 1.453) with reads normalized to 18,517 for each of the cecal sample of broilers (n = 4) fed a basal diet supplemented with 0 (control), 1%, 2% or 4% inulin or 400 ppm bacitracin. The symble labels in (A) apply to (B) and (C).
(PDF)

S2 Fig Pair comparison of the 16S rRNA abundances of top 50 abundant bacterial genera (taxa) identified in the cecal microbiota of chicken fed a basal diet supplemented with 0 (control), 1%, 2% or 4% inulin or 400 ppm bacitracin. (A) Bacitracin treated group vs Control group; (B) 1% inulin treated group vs Control group; (C) 2% inulin treated group vs Control group; (D) 4% inulin treated group vs Control group. *P* values were determined with Kruskal Wallis test. Symbol labels in (A) also apply to (B), (C) and (D).
(PDF)

S1 Table Relative abundance of bacterial genera (taxa) identified in the cecum of broiler chickens fed a corn-based diet supplemented with 0 (control), 1%, 2%, or 4% inulin or 400 ppm bacitracin. (PDF)

S2 Table Relative expression abundance of assigned proteins in the cecal microbiota of broiler chickens fed a corn-based diet supplemented with 0 (control), 1%, 2% or 4% inulin or 400 ppm bacitracin. (PDF)

S3 Table Bacterial relative expression abundance of family 32 glycoside hydrolases in the meta-transcriptome of the cecal microbiota of chickens fed a basal diet supplemented with 0 (control), 1%, 2% or 4% inulin or 400 ppm bacitracin. (PDF)
