# Supplementary material for: Dietary inulin supplementation modulates the composition and activities of carbohydrate-metabolizing organisms in the cecal microbiota of broiler chickens
Source: PLoS One. 2021 Oct 21;16(10):e0258663. doi: 10.1371/journal.pone.0258663 (PMC8530302; doi:10.1371/journal.pone.0258663)
Supplement: S1 Fig — Rarefaction curves for OTU (Chao1), Shannon index (B) and Coverage ratios (C) calculated using Mothur (v 1.453) with reads normalized to 18,517 for each of the cecal sample of broilers (n = 4) fed a basal diet supplemented with 0 (control), 1%, 2% or 4% inulin or 400 ppm bacitracin. The symble labels in (A) apply to (B) and (C). (PDF) [file pone.0258663.s001.pdf]

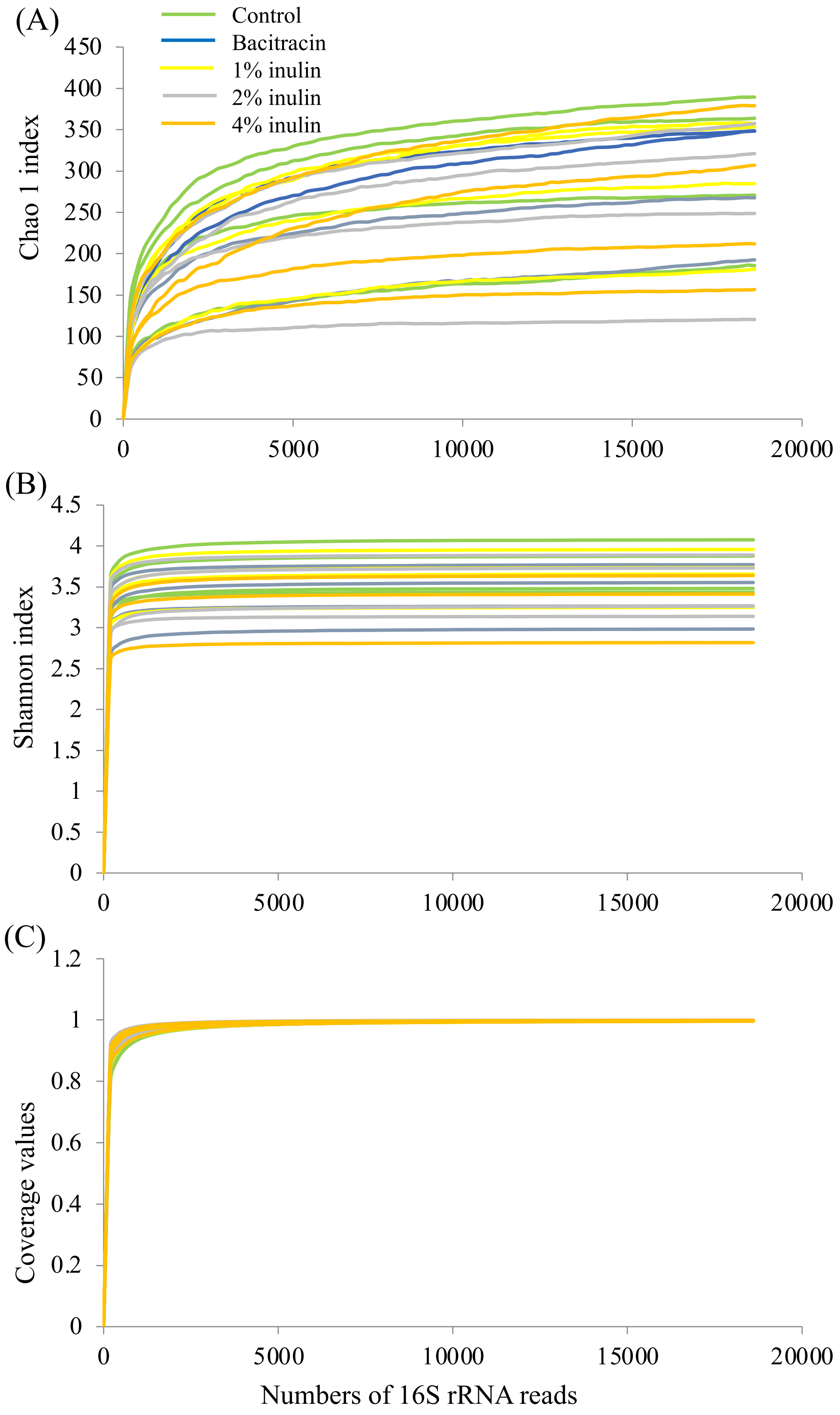

S1 Fig. Rarefaction curves for OTU (Chao1), Shannon index (B) and Coverage values (C) calculated using Mothur (v 1.453) with reads normalized to 18,517 for each of the cecal sample of broilers (n = 4) fed a basal diet supplemented with 0 (control), 1%, 2% or 4% inulin or 400 ppm bacitracin. The symble labels in (A) apply to (B) and (C).
