# Supplementary material for: Dietary inulin supplementation modulates the composition and activities of carbohydrate-metabolizing organisms in the cecal microbiota of broiler chickens
Source: PLoS One. 2021 Oct 21;16(10):e0258663. doi: 10.1371/journal.pone.0258663 (PMC8530302; doi:10.1371/journal.pone.0258663)
Supplement: S2 Fig — (A) Bacitracin treated group vs Control group; (B) 1% inulin treated group vs Control group; (C) 2% inulin treated group vs Control group; (D) 4% inulin treated group vs Control group. P values were determined with Kruskal Wallis test. Symbol labels in (A) also apply to (B), (C) and (D). (PDF) [file pone.0258663.s002.pdf]

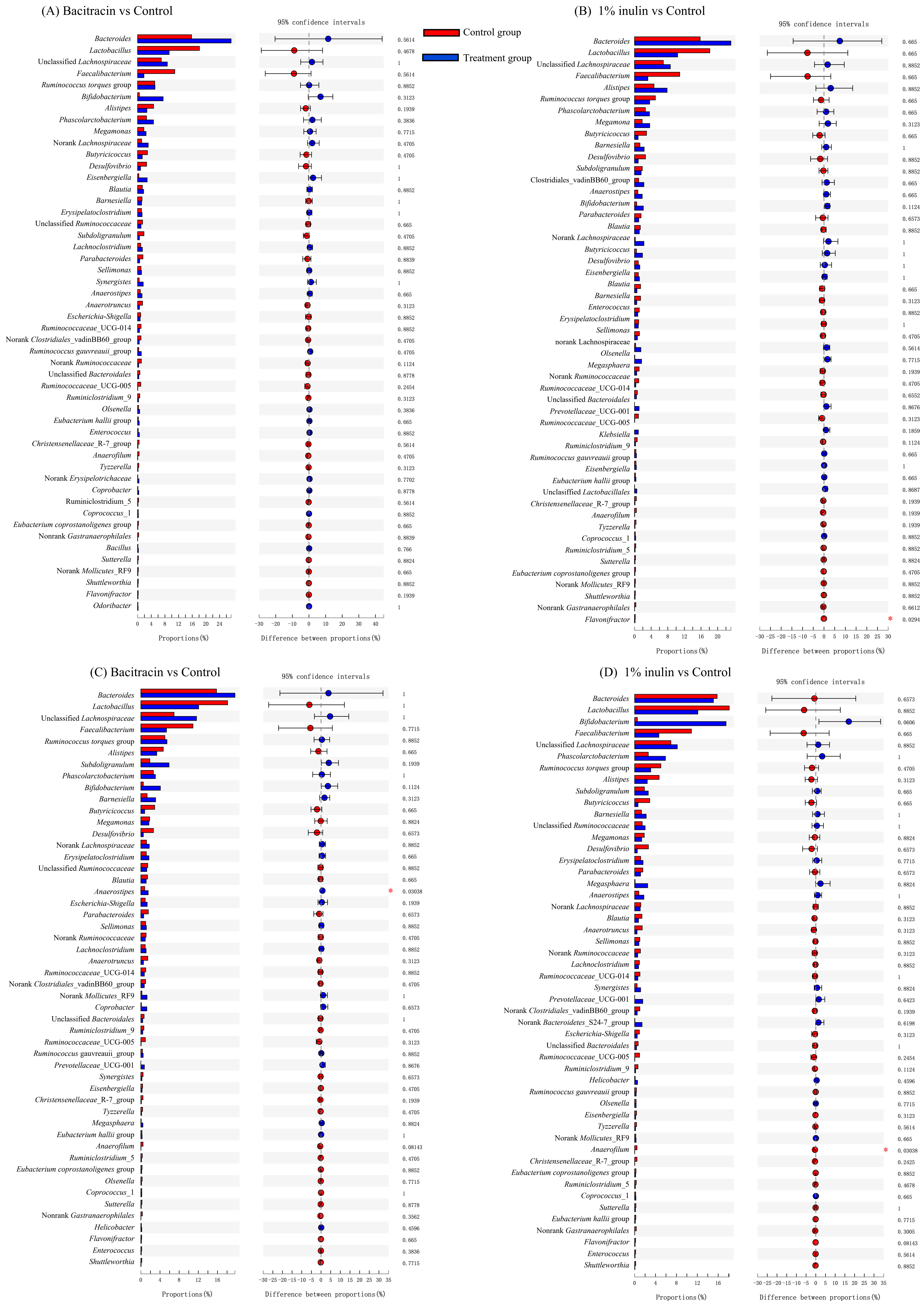

S2 Fig. Pair comparison of the 16S rRNA abundances of top 50 abundant bacterial genera (taxa) identified in the cecal microbiota of chicken fed a basal diet supplemented with 0 (control), 1%, 2% or 4% inulin or 400 ppm bacitracin. (A) Bacitracin treated group vs Control group; (B) 1% inulin treated group vs Control group; (C) 2% inulin treated group vs Control group; (D) 4% inulin treated group vs Control group. *P* values were determined with Kruskal Wallis test. Symbol labels in (A) also apply to (B), (C) and (D).
