# Supplementary material for: Dietary inulin supplementation modulates the composition and activities of carbohydrate-metabolizing organisms in the cecal microbiota of broiler chickens
Source: PLoS One. 2021 Oct 21;16(10):e0258663. doi: 10.1371/journal.pone.0258663 (PMC8530302; doi:10.1371/journal.pone.0258663)
Supplement: S1 Table — (PDF) [file pone.0258663.s003.pdf]

S1 Table. Relative 16S rRNA abundance of bacterial genera identified in the cecum of broiler chickens fed a corn-based diet supplemented with 0 (control), 1%, 2%, or 4% inulin or 400 ppm bacitracin.

Genus (taxa) names	Conotrol		Bacitracin		1% inulin		2% inulin		4% inulin	
	Mean[a]	STD[b]	Mean	STD	Mean	STD	Mean	STD	Mean	STD
Bacteroides	15.79%	17.63%	27.37%	29.35%	23.18%	15.40%	19.64%	22.82%	15.08%	17.23%
Lactobacillus	18.11%	18.12%	9.24%	10.12%	10.36%	10.60%	12.06%	10.74%	12.10%	11.81%
unclassified *Lachnospiraceae*	6.93%	2.95%	8.70%	6.88%	8.61%	6.94%	11.62%	8.91%	8.16%	5.90%
Bifidobacterium	0.51%	0.82%	7.47%	7.95%	2.11%	0.89%	4.07%	4.63%	17.50%	16.59%
Faecalibacterium	10.87%	15.88%	1.85%	1.99%	3.21%	2.75%	5.35%	3.69%	4.63%	6.75%
[*Ruminococcus*]_torques_group	5.01%	3.68%	5.09%	4.42%	3.64%	0.85%	5.45%	2.51%	3.07%	1.44%
Alistipes	4.68%	2.82%	2.71%	1.61%	7.83%	9.26%	3.32%	3.14%	2.41%	1.81%
Phascolarctobacterium	2.61%	3.23%	4.65%	4.95%	3.60%	2.69%	3.03%	3.73%	5.90%	8.99%
Subdoligranulum	1.86%	1.95%	0.54%	0.36%	1.59%	1.25%	5.88%	4.39%	2.61%	1.70%
Megamonas	1.83%	2.44%	2.47%	3.19%	3.65%	3.91%	1.69%	2.66%	1.34%	1.50%
Barnesiella	1.30%	1.33%	1.17%	1.63%	2.31%	1.98%	3.08%	2.55%	2.21%	2.99%
norank *Lachnospiraceae*	1.18%	0.86%	3.12%	3.75%	0.69%	0.56%	1.76%	1.46%	1.06%	1.13%
Anaerostipes	0.80%	0.13%	1.30%	1.37%	1.85%	1.88%	1.53%	0.47%	1.76%	2.09%
Butyricicoccus	2.88%	3.16%	1.36%	1.58%	0.85%	0.51%	0.79%	0.44%	0.64%	0.48%
Erysipelatoclostridium	1.14%	0.99%	1.31%	1.34%	0.79%	0.35%	1.68%	1.66%	1.61%	2.40%
unclassified *Ruminococcaceae*	1.45%	1.24%	0.99%	0.79%	0.58%	0.20%	1.24%	0.87%	2.01%	2.86%
Blautia	1.40%	0.74%	1.76%	1.78%	1.15%	1.01%	1.11%	0.94%	0.74%	0.47%
Desulfovibrio	2.63%	4.22%	0.86%	1.50%	0.89%	1.39%	0.51%	0.82%	0.50%	0.78%
Synergistes	0.47%	0.52%	1.65%	3.00%	1.91%	3.44%	0.24%	0.43%	1.14%	2.10%
Lachnoclostridium	0.91%	0.61%	1.41%	1.45%	1.18%	1.02%	1.10%	0.90%	0.75%	0.56%
Sellimonas	0.99%	0.88%	1.14%	0.92%	0.91%	0.63%	1.15%	0.93%	0.82%	0.62%
norank *Clostridiales*_vadinBB60_group	0.99%	0.63%	0.49%	0.15%	2.25%	3.26%	0.72%	0.62%	0.49%	0.60%
Parabacteroides	1.57%	2.50%	0.61%	1.09%	1.02%	1.54%	0.59%	0.74%	1.13%	1.74%

Megasphaera	0.08%	0.07%	0.17%	0.29%	1.69%	1.91%	0.43%	0.76%	2.49%	4.60%
Escherichia-Shigella	0.91%	1.57%	0.75%	0.84%	1.27%	2.20%	1.32%	1.99%	0.49%	0.54%
Eisenbergiella	0.36%	0.16%	2.81%	4.67%	0.44%	0.34%	0.26%	0.17%	0.19%	0.11%
Anaerotruncus	1.45%	1.11%	0.56%	0.42%	0.53%	0.37%	0.55%	0.28%	0.46%	0.30%
*Prevotellaceae*_UCG-001	0.00%	0.00%	0.11%	0.13%	1.10%	2.04%	0.74%	1.36%	1.55%	2.86%
norank *Ruminococcaceae*	1.10%	1.09%	0.31%	0.15%	0.52%	0.47%	0.92%	1.01%	0.58%	0.67%
*Ruminococcaceae*_UCG-014	1.02%	1.08%	0.56%	0.48%	0.31%	0.17%	0.73%	0.58%	0.62%	0.46%
Enterococcus	0.18%	0.16%	0.52%	0.72%	2.26%	4.09%	0.10%	0.16%	0.08%	0.09%
Olsenella	0.28%	0.27%	0.59%	0.48%	1.52%	1.66%	0.14%	0.12%	0.28%	0.47%
*Ruminococcus gauvreauii*_group	0.34%	0.29%	1.08%	1.08%	0.46%	0.41%	0.48%	0.56%	0.29%	0.27%
Unclassified *Bacteroidales*	0.68%	1.08%	0.36%	0.62%	0.46%	0.30%	0.38%	0.42%	0.36%	0.39%
norank *Mollicutes*_RF9	0.22%	0.21%	0.11%	0.12%	0.12%	0.10%	1.29%	2.07%	0.27%	0.18%
Coprobacter	0.11%	0.18%	0.35%	0.57%	0.06%	0.07%	1.25%	2.20%	0.04%	0.05%
*Ruminiclostridium*_9	0.64%	0.41%	0.28%	0.26%	0.24%	0.10%	0.41%	0.31%	0.18%	0.13%
norank *Bacteroidales*_S24-7_group	0.04%	0.08%	0.00%	0.00%	0.00%	0.01%	0.11%	0.21%	1.41%	2.61%
Eubacterium hallii group	0.23%	0.25%	0.49%	0.61%	0.35%	0.17%	0.27%	0.21%	0.13%	0.10%
*Ruminococcaceae*_UCG-005	0.94%	1.50%	0.05%	0.03%	0.04%	0.05%	0.06%	0.03%	0.04%	0.06%
*Coprococcus*_1	0.19%	0.16%	0.22%	0.13%	0.27%	0.30%	0.19%	0.07%	0.24%	0.17%
Klebsiella	0.00%	0.00%	0.14%	0.26%	0.92%	1.69%	0.02%	0.02%	0.00%	0.00%
Helicobacter	0.11%	0.19%	0.08%	0.12%	0.08%	0.05%	0.19%	0.22%	0.55%	0.66%
Tyzzerella	0.36%	0.34%	0.14%	0.12%	0.12%	0.05%	0.15%	0.11%	0.17%	0.11%
*Ruminiclostridium*_5	0.31%	0.32%	0.11%	0.08%	0.14%	0.08%	0.13%	0.09%	0.13%	0.11%
Eubacterium coprostanoligenes group	0.26%	0.22%	0.10%	0.04%	0.10%	0.07%	0.17%	0.04%	0.19%	0.07%
*Christensenellaceae*_R-7_group	0.44%	0.52%	0.13%	0.11%	0.12%	0.08%	0.11%	0.09%	0.02%	0.01%
norank *Erysipelotrichaceae*	0.07%	0.03%	0.40%	0.60%	0.06%	0.04%	0.12%	0.08%	0.12%	0.09%
Sutterella	0.24%	0.37%	0.09%	0.10%	0.12%	0.09%	0.10%	0.13%	0.15%	0.16%
unclassified *Lactobacillales*	0.02%	0.03%	0.06%	0.07%	0.55%	1.03%	0.02%	0.04%	0.01%	0.02%
Flavonifractor	0.18%	0.05%	0.13%	0.07%	0.13%	0.01%	0.12%	0.06%	0.08%	0.04%
Anaerofilum	0.45%	0.64%	0.06%	0.05%	0.04%	0.03%	0.03%	0.03%	0.01%	0.01%

Shuttleworthia	0.20%	0.24%	0.12%	0.07%	0.14%	0.11%	0.06%	0.04%	0.05%	0.03%
Odoribacter	0.10%	0.09%	0.15%	0.17%	0.05%	0.04%	0.11%	0.19%	0.09%	0.10%
unclassified *Selenomonadales*	0.07%	0.10%	0.12%	0.13%	0.10%	0.11%	0.07%	0.13%	0.10%	0.11%
unclassified *Firmicutes*	0.07%	0.11%	0.09%	0.11%	0.06%	0.09%	0.10%	0.14%	0.11%	0.16%
norank *Gastranaerophilales*	0.32%	0.56%	0.04%	0.04%	0.02%	0.02%	0.02%	0.04%	0.01%	0.01%
norank *Porphyromonadaceae*	0.06%	0.06%	0.08%	0.09%	0.13%	0.15%	0.06%	0.09%	0.06%	0.08%
Ruminococcaceae_UCG-004	0.09%	0.04%	0.06%	0.05%	0.11%	0.07%	0.09%	0.09%	0.04%	0.02%
Bacillus	0.11%	0.15%	0.23%	0.43%	0.01%	0.02%	0.01%	0.02%	0.01%	0.01%
Butyricimonas	0.19%	0.13%	0.05%	0.05%	0.06%	0.08%	0.02%	0.03%	0.03%	0.05%
*Ruminococcaceae*_NK4A214_group	0.12%	0.09%	0.11%	0.08%	0.04%	0.03%	0.04%	0.04%	0.02%	0.02%
unclassified *Porphyromonadaceae*	0.11%	0.13%	0.03%	0.04%	0.03%	0.03%	0.13%	0.18%	0.03%	0.03%
Senegalimassilia	0.06%	0.06%	0.13%	0.12%	0.08%	0.09%	0.04%	0.04%	0.02%	0.02%
Mucispirillum	0.10%	0.13%	0.01%	0.02%	0.08%	0.09%	0.08%	0.12%	0.04%	0.08%
Rikenella	0.16%	0.18%	0.05%	0.08%	0.01%	0.01%	0.03%	0.05%	0.02%	0.04%
unclassified *Clostridiales*	0.09%	0.09%	0.05%	0.05%	0.05%	0.05%	0.04%	0.06%	0.03%	0.06%
*Ruminococcaceae*_UCG-013	0.06%	0.04%	0.10%	0.11%	0.03%	0.03%	0.04%	0.02%	0.04%	0.04%
Bilophila	0.05%	0.06%	0.04%	0.07%	0.05%	0.06%	0.05%	0.09%	0.04%	0.06%
Enorma	0.02%	0.02%	0.01%	0.02%	0.04%	0.04%	0.08%	0.11%	0.05%	0.06%
Caproiciproducens	0.05%	0.04%	0.02%	0.01%	0.01%	0.01%	0.01%	0.01%	0.11%	0.18%
*Ruminococcaceae*_UCG-010	0.11%	0.18%	0.04%	0.02%	0.02%	0.02%	0.01%	0.01%	0.01%	0.01%
Candidatus *Arthromitus*	0.03%	0.05%	0.06%	0.11%	0.01%	0.02%	0.03%	0.03%	0.03%	0.04%
unclassified *Coriobacteriaceae*	0.02%	0.02%	0.02%	0.03%	0.07%	0.07%	0.04%	0.07%	0.00%	0.00%
*Tyzzerella*_3	0.03%	0.03%	0.02%	0.01%	0.02%	0.02%	0.03%	0.02%	0.04%	0.04%
Marvinbryantia	0.04%	0.03%	0.02%	0.02%	0.06%	0.07%	0.01%	0.01%	0.01%	0.01%
Succinatimonas	0.01%	0.01%	0.06%	0.07%	0.02%	0.03%	0.02%	0.03%	0.02%	0.03%
norank *Rhodospirillaceae*	0.03%	0.03%	0.02%	0.03%	0.01%	0.03%	0.05%	0.08%	0.01%	0.01%
Campylobacter	0.03%	0.05%	0.01%	0.02%	0.01%	0.02%	0.05%	0.04%	0.02%	0.04%
unclassified *Erysipelotrichaceae*	0.05%	0.06%	0.02%	0.02%	0.01%	0.01%	0.02%	0.01%	0.01%	0.01%
Peptoclostridium	0.08%	0.14%	0.02%	0.04%	0.00%	0.01%	0.00%	0.00%	0.00%	0.00%

Akkermansia	0.01%	0.01%	0.02%	0.04%	0.06%	0.11%	0.01%	0.01%	0.00%	0.00%
*Ruminococcus*_1	0.06%	0.09%	0.03%	0.05%	0.00%	0.00%	0.00%	0.00%	0.00%	0.00%
Roseburia	0.05%	0.04%	0.01%	0.01%	0.02%	0.02%	0.01%	0.01%	0.00%	0.00%
norank *Peptococcaceae*	0.03%	0.04%	0.01%	0.01%	0.03%	0.03%	0.01%	0.01%	0.00%	0.00%
*Ruminococcaceae*_UCG-009	0.02%	0.01%	0.01%	0.01%	0.02%	0.01%	0.02%	0.03%	0.01%	0.01%
Faecalicoccus	0.00%	0.00%	0.00%	0.00%	0.04%	0.06%	0.02%	0.04%	0.00%	0.00%
Elusimicrobium	0.03%	0.05%	0.02%	0.05%	0.01%	0.02%	0.00%	0.00%	0.00%	0.00%
*Coriobacteriaceae*_UCG-002	0.01%	0.01%	0.00%	0.00%	0.04%	0.04%	0.01%	0.01%	0.01%	0.03%
Family_XIII_AD3011_group	0.01%	0.02%	0.01%	0.01%	0.01%	0.01%	0.01%	0.01%	0.02%	0.01%
norank *Flavobacteriaceae*	0.01%	0.01%	0.00%	0.00%	0.01%	0.02%	0.02%	0.03%	0.01%	0.02%
Eubacterium nodatum group	0.01%	0.01%	0.01%	0.01%	0.02%	0.02%	0.01%	0.01%	0.01%	0.02%
Peptococcus	0.02%	0.04%	0.00%	0.00%	0.01%	0.02%	0.00%	0.01%	0.01%	0.03%
Anaerofustis	0.02%	0.03%	0.00%	0.00%	0.01%	0.01%	0.00%	0.00%	0.01%	0.01%
norank WCHB1-41	0.01%	0.01%	0.00%	0.01%	0.04%	0.07%	0.00%	0.00%	0.00%	0.00%
Eubacterium	0.01%	0.01%	0.00%	0.01%	0.00%	0.01%	0.02%	0.04%	0.01%	0.01%
Eubacterium brachy group	0.00%	0.00%	0.00%	0.00%	0.01%	0.01%	0.01%	0.01%	0.01%	0.01%
*Lachnospiraceae*_FCS020_group	0.00%	0.00%	0.01%	0.01%	0.01%	0.00%	0.01%	0.01%	0.00%	0.00%
*Clostridium sensu*_stricto_1	0.02%	0.03%	0.00%	0.00%	0.00%	0.00%	0.00%	0.00%	0.01%	0.02%
unclassified Family_XIII	0.01%	0.01%	0.01%	0.00%	0.01%	0.00%	0.01%	0.01%	0.01%	0.00%
Collinsella	0.01%	0.02%	0.00%	0.00%	0.01%	0.01%	0.01%	0.02%	0.00%	0.00%
*Lachnospiraceae*_UCG-010	0.01%	0.01%	0.01%	0.01%	0.00%	0.00%	0.01%	0.01%	0.01%	0.00%
norank *Cyanobacteria*	0.00%	0.00%	0.00%	0.00%	0.03%	0.03%	0.00%	0.00%	0.00%	0.00%
unclassified *Peptococcaceae*	0.02%	0.04%	0.00%	0.00%	0.00%	0.00%	0.00%	0.00%	0.00%	0.01%
Intestinimonas	0.01%	0.01%	0.00%	0.00%	0.01%	0.02%	0.00%	0.00%	0.00%	0.00%
*Ruminococcus*_2	0.02%	0.02%	0.00%	0.01%	0.00%	0.01%	0.00%	0.00%	0.00%	0.00%
*Defluviitaleaceae*_UCG-011	0.00%	0.00%	0.01%	0.02%	0.00%	0.00%	0.01%	0.01%	0.00%	0.01%
Eubacterium ventriosum group	0.01%	0.02%	0.00%	0.00%	0.00%	0.00%	0.00%	0.00%	0.00%	0.00%
*Ruminiclostridium*_1	0.01%	0.01%	0.00%	0.01%	0.01%	0.01%	0.00%	0.00%	0.00%	0.00%
Family_XIII_UCG-001	0.01%	0.01%	0.00%	0.00%	0.00%	0.00%	0.01%	0.01%	0.00%	0.00%
Clostridium innocuum group	0.00%	0.00%	0.00%	0.01%	0.00%	0.00%	0.01%	0.01%	0.00%	0.00%

Candidatus Soleaferrea	0.01%	0.02%	0.00%	0.00%	0.00%	0.00%	0.00%	0.00%	0.00%	0.00%
Catabacter	0.00%	0.00%	0.00%	0.00%	0.00%	0.00%	0.00%	0.01%	0.00%	0.00%
Streptococcus	0.00%	0.00%	0.01%	0.02%	0.00%	0.00%	0.00%	0.01%	0.00%	0.00%
norank *Victivallaceae*	0.00%	0.00%	0.01%	0.01%	0.01%	0.01%	0.00%	0.00%	0.00%	0.00%
unclassified *Prevotellaceae*	0.00%	0.00%	0.00%	0.00%	0.00%	0.00%	0.01%	0.02%	0.00%	0.00%
Tenacibaculum	0.00%	0.00%	0.00%	0.00%	0.01%	0.02%	0.00%	0.00%	0.00%	0.00%
Brevundimonas	0.00%	0.00%	0.00%	0.00%	0.00%	0.00%	0.00%	0.00%	0.00%	0.00%
Holdemania	0.00%	0.00%	0.00%	0.00%	0.00%	0.00%	0.00%	0.00%	0.00%	0.00%
Slackia	0.00%	0.00%	0.00%	0.00%	0.00%	0.00%	0.00%	0.01%	0.00%	0.00%
Stenotrophomonas	0.00%	0.00%	0.00%	0.00%	0.00%	0.00%	0.00%	0.01%	0.00%	0.00%
Alloprevotella	0.00%	0.00%	0.00%	0.00%	0.00%	0.00%	0.00%	0.01%	0.00%	0.00%

[a]Mean of 4 replicates.

[b]STD represents standard deviation of the mean calculated from 4 replicates.
