# Supplementary material for: Dietary inulin supplementation modulates the composition and activities of carbohydrate-metabolizing organisms in the cecal microbiota of broiler chickens
Source: PLoS One. 2021 Oct 21;16(10):e0258663. doi: 10.1371/journal.pone.0258663 (PMC8530302; doi:10.1371/journal.pone.0258663)
Supplement: S2 Table — (PDF) [file pone.0258663.s004.pdf]

S2 Table. Relative expression abundance of assigned proteins in the cecal microbiota of broiler chickens fed a corn-based diet supplemented with 0 (control), 1%, 2% or 4% inulin or 400 ppm bacitracin.

Taxon	Control	Bacitracin	1% inulin	2% inulin	4% inulin
Bacteroides	34.57%	47.07%	46.04%	45.51%	45.13%
Prevotella	0.47%	0.88%	3.67%	15.28%	13.02%
Blastocystis	10.78%	5.15%	3.29%	2.09%	0.69%
Alistipes	3.31%	3.89%	1.81%	3.76%	3.55%
Parabacteroides	5.34%	4.26%	4.64%	2.98%	3.15%
Faecalibacterium	3.72%	1.25%	0.76%	1.73%	3.28%
Clostridium	2.56%	1.81%	2.53%	1.11%	1.18%
Desulfovibrio	2.48%	2.38%	1.13%	1.07%	0.52%
Phascolarctobacterium	0.69%	0.62%	1.05%	2.71%	3.15%
Megamonas	2.43%	1.10%	1.49%	0.90%	1.51%
Helicobacter	0.87%	1.20%	3.76%	1.24%	1.43%
Sutterella	1.32%	1.55%	2.38%	1.53%	0.80%
Norank *Firmicutes*	2.68%	1.76%	0.98%	0.67%	0.80%
Cloacibacillus	0.76%	1.39%	0.97%	0.98%	0.77%
Methanocorpusculum	1.28%	1.15%	1.84%	0.92%	0.75%
Norank *Bacteroidales*	1.22%	1.52%	1.05%	0.61%	0.97%
Norank *Bacteroides*	2.77%	1.25%	1.14%	0.33%	0.43%
Barnesiella	1.12%	0.42%	0.87%	0.71%	1.85%
Blautia	1.55%	0.99%	0.67%	0.79%	1.14%
Norank *Clostridiales*	0.86%	1.83%	0.56%	0.33%	0.63%
Norank *Eubacteriaceae*	1.18%	0.92%	1.21%	0.18%	0.98%
Megasphaera	0.03%	0.04%	1.43%	1.08%	1.33%
Odoribacter	0.31%	0.44%	0.83%	0.58%	1.22%
Ruminococcus	0.63%	0.73%	0.60%	0.60%	0.61%
Succinatimonas	0.24%	1.32%	1.27%	0.17%	0.14%
Subdoligranulum	0.40%	0.16%	1.20%	1.08%	0.48%
Synergistes	0.28%	0.65%	0.35%	0.38%	0.28%
Bifidobacterium	0.02%	0.04%	0.89%	1.59%	0.25%
Eubacterium	0.60%	0.79%	0.38%	0.32%	0.40%
Norank *Clostridia*	1.55%	0.42%	0.18%	0.08%	0.08%
Norank *Synergistaceae*	0.27%	0.49%	0.33%	0.33%	0.25%
Mucispirillum	0.24%	0.69%	0.05%	0.83%	0.49%
Campylobacter	0.44%	0.03%	0.44%	0.46%	0.29%
Alicyclobacillus	0.20%	0.21%	0.33%	0.44%	0.49%
Norank *Lachnospiraceae*	0.41%	0.43%	0.30%	0.25%	0.32%
Lachnoclostridium	0.40%	0.35%	0.23%	0.25%	0.19%
Norank *Methanomicrobiales*	0.25%	0.00%	1.16%	0.00%	0.03%

Bilophila	0.32%	0.69%	0.12%	0.16%	0.08%
Rotavirus	0.00%	0.16%	0.00%	0.05%	0.89%
Lactobacillus	0.35%	0.51%	0.35%	0.07%	0.08%
Norank bacteria	0.30%	0.23%	0.24%	0.17%	0.24%
Gallus	0.14%	0.07%	0.14%	0.20%	0.69%
Norank *Ruminococcaceae*	0.53%	0.18%	0.18%	0.11%	0.12%
Oscillibacter	0.28%	0.32%	0.18%	0.07%	0.11%
Anaerostipes	0.16%	0.50%	0.11%	0.14%	0.16%
Flavonifractor	0.35%	0.21%	0.19%	0.08%	0.09%
Fusobacterium	0.06%	0.32%	0.47%	0.08%	0.07%
Butyricicoccus	0.19%	0.29%	0.18%	0.06%	0.06%
Dorea	0.17%	0.16%	0.13%	0.18%	0.15%
Tannerella	0.25%	0.12%	0.12%	0.07%	0.13%
Roseburia	0.16%	0.16%	0.18%	0.08%	0.13%
Azospirillum	0.38%	0.24%	0.04%	0.05%	0.02%
Butyricimonas	0.23%	0.16%	0.10%	0.04%	0.14%
Paraprevotella	0.09%	0.19%	0.11%	0.09%	0.12%
Norank *Verrucomicrobia*	0.18%	0.47%	0.00%	0.00%	0.00%
Picobirnavirus	0.00%	0.00%	0.23%	0.30%	0.03%
Acidiphilium	0.08%	0.11%	0.25%	0.09%	0.05%
Ruminiclostridium	0.13%	0.11%	0.13%	0.05%	0.07%
Methanoculleus	0.04%	0.02%	0.23%	0.01%	0.07%
Veillonella	0.07%	0.02%	0.01%	0.02%	0.01%
Acetonema	0.05%	0.05%	0.06%	0.11%	0.17%
Porphyromonas	0.07%	0.08%	0.07%	0.04%	0.09%
Collinsella	0.03%	0.07%	0.06%	0.10%	0.09%
Chlamydia	0.03%	0.04%	0.02%	0.04%	0.01%
Coprobacter	0.14%	0.06%	0.06%	0.04%	0.05%
Candidatus Methanoplasma	0.06%	0.00%	0.26%	0.00%	0.01%
Pseudoflavonifractor	0.14%	0.08%	0.06%	0.03%	0.04%
Fusicatenibacter	0.01%	0.02%	0.05%	0.06%	0.25%
Escherichia	0.08%	0.05%	0.09%	0.05%	0.05%
Olsenella	0.02%	0.05%	0.05%	0.09%	0.09%
Norank *Porphyromonadaceae*	0.15%	0.03%	0.07%	0.02%	0.09%
Dysgonomonas	0.05%	0.02%	0.10%	0.03%	0.05%
Tyzzerella	0.23%	0.03%	0.02%	0.02%	0.02%
Candidatus Methanomethylophilus	0.07%	0.00%	0.21%	0.00%	0.01%
Bacillus	0.08%	0.06%	0.05%	0.09%	0.03%
Hungatella	0.08%	0.05%	0.04%	0.04%	0.08%
Methanoregula	0.06%	0.05%	0.07%	0.04%	0.03%
Macellibacteroides	0.05%	0.08%	0.05%	0.03%	0.04%
Coprococcus	0.05%	0.04%	0.04%	0.08%	0.06%
Anaerotruncus	0.07%	0.05%	0.07%	0.03%	0.03%
Clostridioides	0.08%	0.06%	0.03%	0.03%	0.05%
Methanolinea	0.05%	0.16%	0.02%	0.04%	0.02%

Eggerthella	0.09%	0.09%	0.04%	0.02%	0.02%
Acidaminococcus	0.05%	0.04%	0.08%	0.04%	0.04%
Anaerosporomusa	0.03%	0.03%	0.04%	0.06%	0.09%
Intestinimonas	0.09%	0.07%	0.03%	0.02%	0.03%
Norank *Erysipelotrichaceae*	0.11%	0.05%	0.04%	0.01%	0.02%
Methanobrevibacter	0.00%	0.00%	0.00%	0.16%	0.00%
Paenibacillus	0.06%	0.05%	0.05%	0.03%	0.03%
Akkermansia	0.15%	0.05%	0.00%	0.00%	0.00%
Erysipelatoclostridium	0.11%	0.06%	0.03%	0.01%	0.02%
Acanthamoeba	0.08%	0.01%	0.02%	0.07%	0.04%
Methanoplanus	0.11%	0.00%	0.01%	0.07%	0.04%
Marvinbryantia	0.07%	0.04%	0.03%	0.02%	0.03%
Norank *Bacteroidetes*	0.03%	0.04%	0.03%	0.03%	0.04%
Entamoeba	0.06%	0.01%	0.04%	0.05%	0.03%
Treponema	0.06%	0.08%	0.02%	0.02%	0.02%
Streptococcus	0.03%	0.07%	0.03%	0.03%	0.03%
Atopobium	0.01%	0.03%	0.02%	0.05%	0.05%
Norank *Oscillospiraceae*	0.03%	0.05%	0.05%	0.01%	0.02%
Coraliomargarita	0.12%	0.04%	0.01%	0.02%	0.00%
Dictyostelium	0.06%	0.01%	0.01%	0.05%	0.03%
Acinetobacter	0.02%	0.02%	0.09%	0.01%	0.02%
Brachyspira	0.01%	0.02%	0.10%	0.02%	0.01%
Butyrivibrio	0.05%	0.03%	0.03%	0.02%	0.02%
Gammacoronavirus	0.12%	0.00%	0.00%	0.01%	0.01%
Enterococcus	0.04%	0.03%	0.02%	0.02%	0.02%
Acetobacter	0.11%	0.03%	0.00%	0.00%	0.00%
Norank *Opitutaceae*	0.07%	0.07%	0.00%	0.00%	0.00%
Daphnia	0.08%	0.03%	0.01%	0.01%	0.00%
Aquimarina	0.06%	0.00%	0.03%	0.02%	0.03%
Streptomyces	0.02%	0.01%	0.03%	0.02%	0.03%
Candidatus Stoquefichus	0.06%	0.04%	0.01%	0.01%	0.01%
Rikenella	0.05%	0.03%	0.01%	0.01%	0.01%
Senegalimassilia	0.05%	0.06%	0.01%	0.01%	0.00%
Norank *Bacteroidaceae*	0.02%	0.01%	0.02%	0.03%	0.04%
Burkholderia	0.04%	0.03%	0.02%	0.01%	0.01%
Corallococcus	0.02%	0.02%	0.05%	0.01%	0.01%
Mastigamoeba	0.04%	0.01%	0.01%	0.04%	0.02%
Sanguibacteroides	0.06%	0.01%	0.01%	0.00%	0.02%
Methanomicrobium	0.02%	0.02%	0.02%	0.02%	0.02%
Pseudomonas	0.02%	0.03%	0.02%	0.02%	0.01%
Norank *Deltaproteobacteria*	0.02%	0.04%	0.01%	0.01%	0.01%
Norank *Coriobacteriaceae*	0.04%	0.05%	0.00%	0.01%	0.01%
Proteiniphilum	0.02%	0.01%	0.01%	0.01%	0.02%
Eisenbergiella	0.03%	0.01%	0.01%	0.02%	0.02%
Norank *Proteobacteria*	0.02%	0.03%	0.02%	0.02%	0.01%

Flavobacterium	0.01%	0.01%	0.01%	0.02%	0.02%
Desulfotomaculum	0.01%	0.06%	0.01%	0.00%	0.00%
Avastrovirus	0.02%	0.00%	0.00%	0.05%	0.00%
Mucinivorans	0.02%	0.01%	0.02%	0.02%	0.02%
Slackia	0.03%	0.05%	0.01%	0.01%	0.00%
Tolypothrix	0.02%	0.03%	0.02%	0.01%	0.01%
Polysphondylium	0.03%	0.00%	0.01%	0.02%	0.02%
Dialister	0.00%	0.00%	0.01%	0.06%	0.01%
Coprobacillus	0.03%	0.01%	0.03%	0.00%	0.01%
Lentimicrobium	0.00%	0.00%	0.00%	0.07%	0.01%
Desulfosporosinus	0.01%	0.04%	0.01%	0.00%	0.00%
Enorma	0.01%	0.01%	0.01%	0.03%	0.02%
Robinsoniella	0.03%	0.01%	0.01%	0.01%	0.01%
Norank *Thermoanaerobacterales*	0.05%	0.01%	0.00%	0.00%	0.01%
Desulfonatronum	0.01%	0.02%	0.01%	0.01%	0.01%
Capnocytophaga	0.01%	0.01%	0.01%	0.02%	0.02%
Phocaeicola	0.01%	0.01%	0.02%	0.01%	0.03%
Buceros	0.05%	0.01%	0.00%	0.01%	0.00%
Desulfomicrobium	0.03%	0.01%	0.01%	0.01%	0.01%
Methanocalculus	0.01%	0.01%	0.02%	0.01%	0.01%
Levivirus	0.00%	0.00%	0.00%	0.00%	0.00%
Sphingobacterium	0.02%	0.01%	0.01%	0.01%	0.01%
Meleagris	0.01%	0.00%	0.01%	0.01%	0.04%
Coturnix	0.01%	0.00%	0.01%	0.01%	0.04%
Norank *Enterobacteriaceae*	0.01%	0.01%	0.01%	0.01%	0.01%
Selenomonas	0.01%	0.01%	0.01%	0.01%	0.01%
Lachnoanaerobaculum	0.01%	0.01%	0.01%	0.01%	0.01%
Gordonibacter	0.02%	0.02%	0.01%	0.02%	0.00%
Elusimicrobium	0.00%	0.02%	0.04%	0.00%	0.00%
Verrucomicrobium	0.02%	0.04%	0.00%	0.00%	0.00%
Norank *Methanomassiliicoccales*	0.01%	0.00%	0.04%	0.00%	0.00%
Acetivibrio	0.02%	0.01%	0.01%	0.01%	0.01%
Acytostelium	0.02%	0.00%	0.00%	0.02%	0.01%
Mycobacterium	0.03%	0.01%	0.02%	0.00%	0.00%
Oribacterium	0.02%	0.02%	0.01%	0.00%	0.00%
Norank *Burkholderiales*	0.01%	0.02%	0.02%	0.01%	0.00%
Shigella	0.01%	0.01%	0.01%	0.01%	0.01%
