# Supplementary material for: Dietary inulin supplementation modulates the composition and activities of carbohydrate-metabolizing organisms in the cecal microbiota of broiler chickens
Source: PLoS One. 2021 Oct 21;16(10):e0258663. doi: 10.1371/journal.pone.0258663 (PMC8530302; doi:10.1371/journal.pone.0258663)
Supplement: S3 Table — (PDF) [file pone.0258663.s005.pdf]

S3 Table. Bacterial relative expression abundance of family 32 glycoside hydrolases in the meta-transcriptome of the cecal microbiota of chickens fed a basal diet supplemented with 0 (control), 1%, 2% or 4% inulin or 400 ppm bacitracin.

No.	Bacterial species	Relative expression abundances (×1000) of family 32 glycoside hydrolases				
		Control	400 ppm Bacitracin	1% inulin	2% inulin	4% inulin
1	*Acholeplasma_axanthum*	0.48%	0.06%	0.00%	0.01%	0.00%
2	*Bacteroides barnesiae*	0.08%	0.02%	2.64%	0.00%	0.53%
3	*Bacteroides clarus*	0.00%	0.00%	0.00%	2.02%	0.05%
4	*Bacteroides_coprocola*	0.42%	1.11%	0.46%	0.68%	0.15%
5	*Bacteroides coprocola*	0.10%	0.40%	0.10%	0.14%	0.00%
6	*Bacteroides coprophilus*_CAG	0.47%	0.05%	0.09%	0.00%	0.01%
7	*Bacteroides_faecichinchillae*	0.42%	0.00%	0.06%	0.00%	0.11%
8	*Bacteroides fragilis*	0.25%	0.00%	0.68%	0.46%	0.08%
9	*Bacteroides_ovatus*	0.50%	0.01%	0.00%	0.00%	0.00%
10	*Bacteroides ovatus*	0.48%	0.00%	0.00%	0.00%	0.00%
11	*Bacteroides plebeius*	0.00%	0.14%	0.00%	0.00%	0.03%
12	*Bacteroides_plebeius*	0.92%	1.46%	0.21%	0.59%	0.12%
13	*Bacteroides plebeius*	0.87%	0.06%	0.46%	0.02%	0.15%
14	*Bacteroides plebeius*	2.00%	0.21%	0.00%	0.00%	0.14%
15	*Bacteroides_salanitronis*	0.00%	0.00%	1.66%	0.00%	0.50%
16	*Bacteroides salanitronis*	5.92%	0.34%	14.57%	4.95%	1.75%
17	*Bacteroides*_sp._CAG	0.63%	1.02%	1.96%	0.01%	0.59%
18	*Bacteroides* sp._CAG	0.72%	0.60%	18.54%	8.76%	4.79%
19	*Bacteroides* sp. CAG	0.43%	2.98%	0.74%	0.70%	0.52%
20	*Bacteroides*_sp._CAG	0.56%	0.00%	0.12%	0.00%	0.00%
21	*Barnesiella viscericola*	0.12%	0.14%	0.59%	1.07%	0.91%
22	*Barnesiella viscericola*	0.11%	0.13%	0.35%	0.90%	0.69%
23	*Bifidobacterium_pullorum*	0.00%	0.00%	0.05%	0.45%	0.04%
24	*Bifidobacterium saeculare*	0.00%	0.00%	0.24%	0.63%	0.00%
25	*Bifidobacterium_saeculare*	0.00%	0.00%	0.72%	2.08%	0.04%
26	*Blautia* sp._Marseille-P2398	0.35%	0.04%	0.00%	0.00%	0.00%
27	Norank *Clostridiales*	0.00%	0.00%	0.17%	0.13%	0.82%
28	*Clostridium_indolis*	0.00%	0.00%	0.00%	0.02%	0.23%
29	*Clostridium* sp._KNHs205	0.09%	0.02%	0.17%	0.16%	0.61%
30	*Eubacterium plexicaudatum*	0.00%	0.02%	0.04%	0.09%	0.48%
31	*Eubacterium*_sp._ER2	0.00%	0.07%	0.81%	0.04%	0.01%
32	*Eubacterium* sp. ER2	0.00%	0.03%	0.05%	0.00%	1.93%
33	*Faecalibacterium_prausnitzii*	0.40%	0.00%	0.00%	0.00%	0.02%

34	*Firmicutes bacterium* ASF500	0.01%	0.01%	0.11%	1.69%	4.53%
35	*Firmicutes*_bacterium_CAG	0.00%	0.00%	0.35%	0.06%	0.00%
36	*Firmicutes* bacterium CAG	0.01%	0.05%	0.06%	0.53%	0.43%
37	*Lachnospiraceae* bacterium_A2	1.12%	1.38%	2.23%	0.05%	0.79%
38	*Lactobacillus*_sp.	0.00%	0.00%	0.33%	0.00%	0.02%
39	*Prevotella denticola*	0.00%	0.00%	1.73%	0.57%	4.00%
40	*Prevotella* sp._CAG	0.00%	0.00%	0.21%	0.00%	0.30%
41	*Prevotella* sp._CAG	0.00%	0.00%	0.26%	0.21%	0.94%
42	*Prevotella* sp._CAG	0.00%	0.00%	1.21%	0.39%	3.26%
43	*Prevotella*_sp._CAG	0.00%	0.00%	0.00%	10.21%	0.04%
44	*Ruminococcaceae* bacterium_AM2	0.00%	0.00%	0.00%	1.42%	0.47%
45	*Ruminococcaceae*_bacterium_AM2	0.00%	0.00%	2.01%	0.12%	0.01%
46	*Ruminococcaceae*_bacterium_D16	0.47%	0.44%	0.13%	0.02%	0.20%
47	Uncultured bacterium	0.03%	0.00%	0.00%	0.68%	0.30%
48	Uncultured *Clostridium* sp.	0.00%	0.00%	0.00%	0.06%	0.47%
49	Unclassified bacterium	0.00%	0.00%	0.14%	0.00%	0.31%
50	Unclassified bacterium	0.06%	0.54%	0.17%	0.09%	0.00%
51	Unclassified bacterium	0.00%	0.00%	0.27%	0.00%	0.10%
52	Unclassified bacterium	0.00%	0.00%	0.00%	0.00%	0.08%
53	Unclassified bacterium	0.38%	0.00%	0.08%	0.00%	0.00%
54	Unclassified bacterium	0.12%	0.00%	0.12%	0.00%	0.00%
55	Unclassified bacterium	0.16%	0.05%	0.06%	0.00%	0.05%
56	Unclassified bacterium	0.00%	0.27%	0.00%	0.00%	0.00%
57	Unclassified bacterium	0.00%	0.00%	0.24%	0.04%	0.00%
58	Unclassified bacterium	0.04%	0.19%	0.16%	0.23%	0.03%
59	Unclassified bacterium	0.00%	0.00%	0.00%	0.17%	0.00%
60	Unclassified bacterium	0.15%	0.24%	0.00%	0.00%	0.00%
61	Unclassified bacterium	0.28%	0.10%	0.15%	0.04%	0.07%
62	Unclassified bacterium	0.05%	0.07%	0.13%	0.23%	0.23%
63	Unclassified bacterium	0.00%	0.21%	0.00%	0.00%	0.00%
64	Unclassified bacterium	0.04%	0.17%	0.05%	0.00%	0.04%
65	Unclassified bacterium	0.13%	0.00%	0.00%	0.00%	0.00%
66	Unclassified bacterium	0.05%	0.00%	0.43%	0.07%	0.02%
67	Unclassified bacterium	0.00%	0.00%	0.00%	0.49%	0.00%
68	Unclassified bacterium	0.05%	0.00%	0.14%	0.00%	0.00%
